# Observations on a Case of Small-Pox Having Supervened after the Vaccine Inoculation

**Published:** 1800-10

**Authors:** A. Huggan

**Affiliations:** Tenterden, Kent


### *Observations on a Case of Small-pox having supervened after the Vaccine Inoculation; by* DR. HUGGAN.

To the EDITORS of the MEDICAL and PHYSICAL JOURNAL.

Gentlemen,

WE find, in the 17th Number of the London Medical Review and Magazine, p. 105, a Cafe inferted "of Small-pox having fupervened after the Vaccine Inoculation," which the Author tells us, has "been the fubject of much converfation," and "fome mifreprefentation;" and left any perfon fhould doubt this ftatement, in the fubfequent number, to wit, the

 laft, laft, p. 208, we find a fecond letter on the fame cafe, wherein the Author affures us, "that there can no longer remain a doubt upon that fubject; for," fays he, "I have inoculated with the matter, and produced Small-pox in the moft fatisfactory way."

That this Cafe fhould have occafioned "much converfation" amongft thofe who were *weak* enough to believe fuch an occurrence *probable*, or even *poffible*, can eafily be fuppofed; and that it has alfo been the fubject of "fome" (nay much) "mis-reprefentation," few will doubt, who have confidered the cafe as related by the Author himfelf, and which we fhall now examine more particularly. With regard to the difeafe that immediately followed the Vaccine Inoculation, "On the third day," we are told, that "there were fufficient marks of the action of the virus;" and that "on the eighth day, the child complained of head-ach and *fickness*, had a quick pulfe, *white tongue*, and increafed heat, with an *enlargement* and tendernefs of the axillæ."

If I may be allowed to give an opinion from my own experience, from that of my friends which I have feen, and from all that I have read upon the fubject of the Cow-pox, I will venture to affirm, that the Vaccine Inoculation, in fo young a child, has never produced a train of fymptoms either fo violent, or that have followed each other in a fimilar fucceffion, to what we are told took place in this Cafe. No other practitioner, I believe, has ever been able to fay that there were, on the *third* day, *fufficient marks* of the action of the virus. Allowing, however, this to be fometimes the cafe, we fhould have expected, in a fubject fo irritable as this child appears to have been, efpecially from the regular manner, in point of time, with which the fymptoms of the Cow-pox from inoculation are obferved to follow each other, the conftitutional difeafe to have come on, at fartheft, three days afterwards, that is, on the fixth day from the *fuppofed* inoculation with *vaccine* matter; for, I believe, it has been uniformly remarked, that if, on the fifth day after this inoculation, we can, from unequivocal and "fufficient marks of the action of the virus," pronounce infection to have taken place, the conftitutional difeafe commences upon the eighth. Nor do I ever recollect to have feen or heard of the Cow-pox, in infants, being accompanied with *fickness*, *white tongue*, nor even much increafed heat; nor have I ever perceived any *enlargement* in the axilla, though always a tendernefs, efpecially when touched.

To be more circumftantial is unneceffary, as the Cafe fpeaks for itfelf; every medical man, much converfant in difeafe, muft have occafionally obferved a fimilar train of fymptoms to have followed a cut puncture from a foul lancet or other pointed inftrument,

ſtrument, independent of the matter that made it ſo; and there is not a doubt but that this child's *first* diſeaſe was imputable to ſuch a caufe.

With the above Caſe, the Editors of the Magazine in which it appeared, have very properly contraſted a copy of the Teſtimonial, in favour of the Vaccine Inoculation, that has been ſigned by ſo many reſpectable Practitioners in London, and to which, any further individual teſtimony in its favour is *now* quite unneceſſary; and with regard to the purport of the Author's ſecond letter, they have obſerved, that it "does not leſſen the importance of the Vaccine Inoculation,—ſince it ſtill remains a certain preventive of the Small-pox."

It has alſo been elſewhere very juſtly obſerved, (Medical and Chirurgical Review, laſt Number, p. 190) that the "Hiſtory" of this Caſe "is defective, in not deſcribing more minutely the appearances of the inoculated part in its different ſtages." This is certainly a very important point, as on the appearances of it, when other ſymptoms are not perceptible, we are obliged to form our indication, whether or not the whole ſyſtem has been affected.

It is, certainly, now too late of the day for Practitioners to be amuſing themſelves with publiſhing Caſes altogether inconcluſive of what they ſeem deſirous to be inferred from them, or in hopes of being able to controvert the *efficacy* of the Cow-pox, in preſerving the human conſtitution from the infection of the Small-pox. Such caſes, indeed, may impoſe upon the credulous, may perplex the minds of thoſe who ſtill have their doubts on the ſubject, and may afford a malicious and ſhort-lived triumph to the ungenerous part of the Profeſſion, and thoſe who think themſelves intereſted in oppoſing the Vaccine Inoculation; but they never can influence the liberal and enlightened mind, that grounds its concluſions upon the ſteady and uniform reſult of extenſive and deciſive experience.

Magna eſt veritas, et prevalebit.

——————— 'Tis evidence ſo full—

If the laſt trumpet ſounded in my ear,

Undaunted I ſhould meet the Saints half way,

And, in the face of Heav'n, maintain the fact.

DRYDEN.

Now, leſt your ſilence on the Caſe alluded to ſhould be conſtrued in a light different from that in which you have conſidered it, I have taken the liberty of troubling you with theſe few Remarks; which, if you think the formal refutation of ſuch a Caſe at all neceſſary, I will thank you to inſert in the enſuing Number of your Journal. I have to regret that I had it it not in my power to have sent them sooner, as from unforeseen delay I did not receive your last Number till yesterday.

I am, Gentlemen,

Your most obedient servant,

A. HUGGAN.

*Tenterden, Kent,*

